# Supplementary material for: The electric field cavity array effect of 2D nano-sieves
Source: Nat Commun. 2022 Dec 22;13:7887. doi: 10.1038/s41467-022-35623-5 (PMC9780201; doi:10.1038/s41467-022-35623-5)
Supplement: Supplementary file 1 — supporting information [file 41467_2022_35623_MOESM1_ESM.pdf]

Supplementary Information

The electric field cavity array effect of 2D nano-sieves

Fan Xu, Yuke Li, Qing Zou, Yushuang He, Zijia Shen, Chen Li, Huijuan Zhang, Feipeng Wang, Jian Li, Yu Wang*

Supplementary Note 1. Co_3O_4 nano-materials structure and morphology characterization

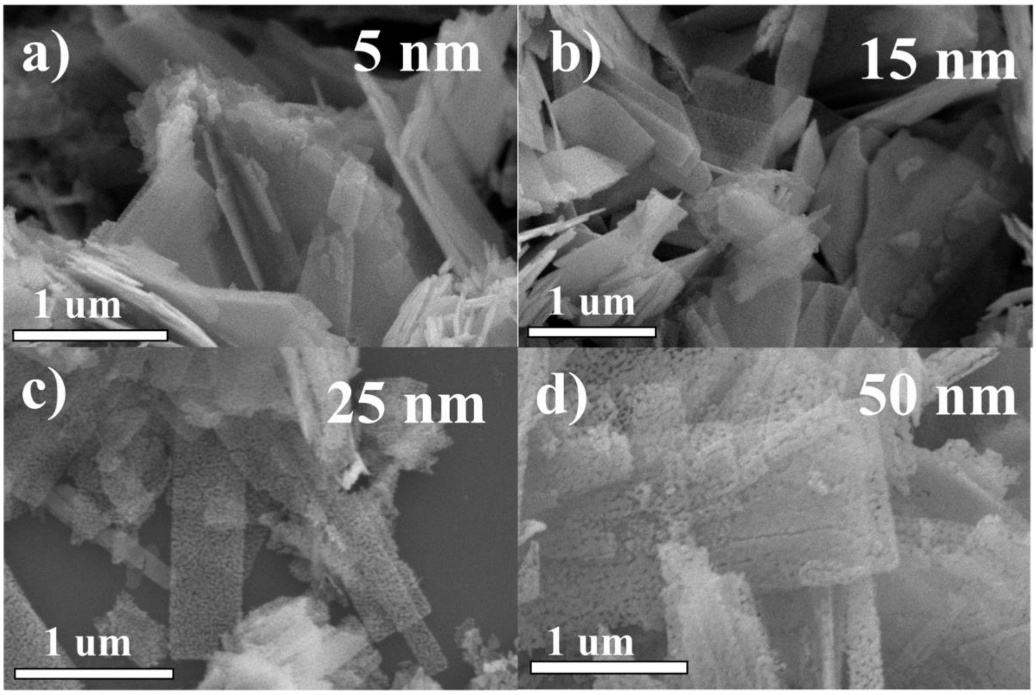

Supplementary Figure 1. Low magnification scanning electron microscope (SEM) of Co_3O_4 nano-sieves (NSIs) with (a) 5 nm, (b) 15 nm, (c) 25 nm, (d) 50nm pore size.

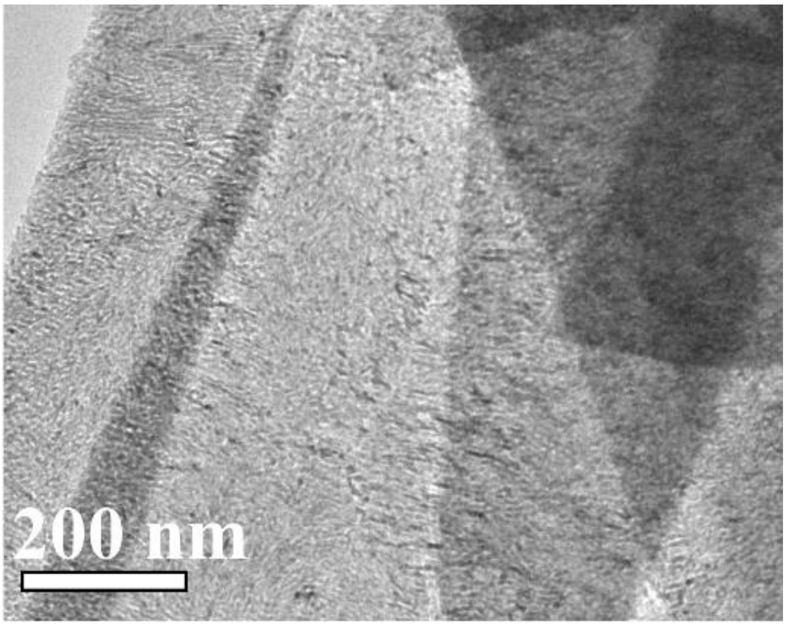

Supplementary Figure 2. Transmission electron microscopy (TEM) image of Co_3O_4 NSIs

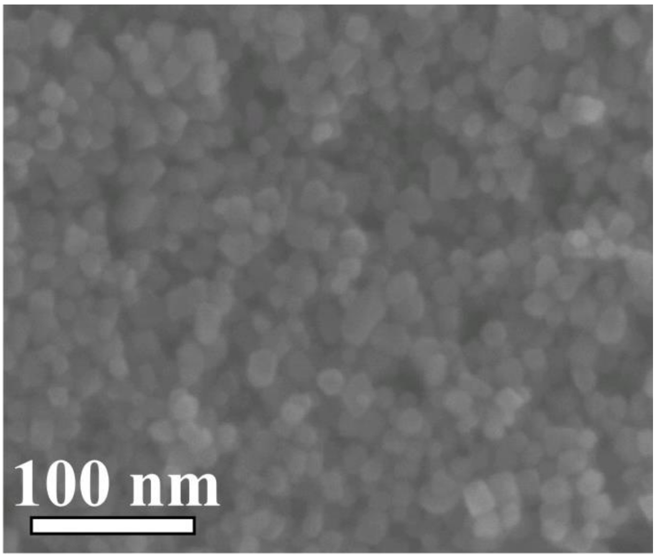

Supplementary Figure 3. SEM of Co_3O_4 nanoparticles (NPs) with a mean size of 16 nm.

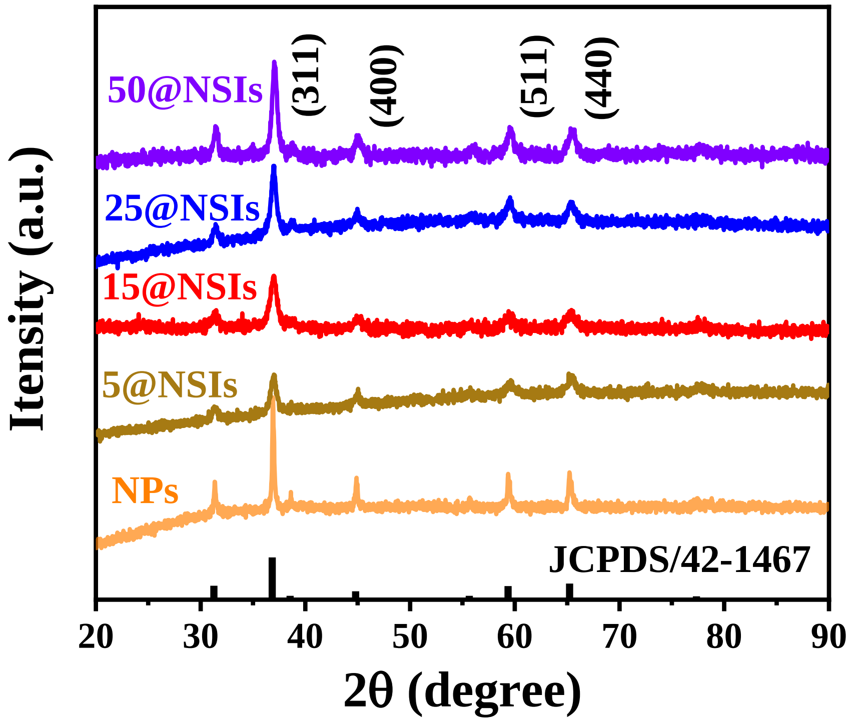

Supplementary Figure 4. X-ray diffraction (XRD) patterns of Co_3O_4 NPs, NSIs with 5 nm pore size (denoted as 5@NSIs), 15@NSIs, 25@NSIs, 50@NSIs. All the diffraction peaks of XRD are well-indexed to face-centered cubic (fcc) Co_3O_4 in JCPDS Card No. 42-1467 with a lattice constant of a=0.8084 nm. The characteristic reflections at ~31.3° and ~36.8° are assigned to the (220) and (311) plane of Co_3O_4 nanocrystals, consistent with HRTEM result.2

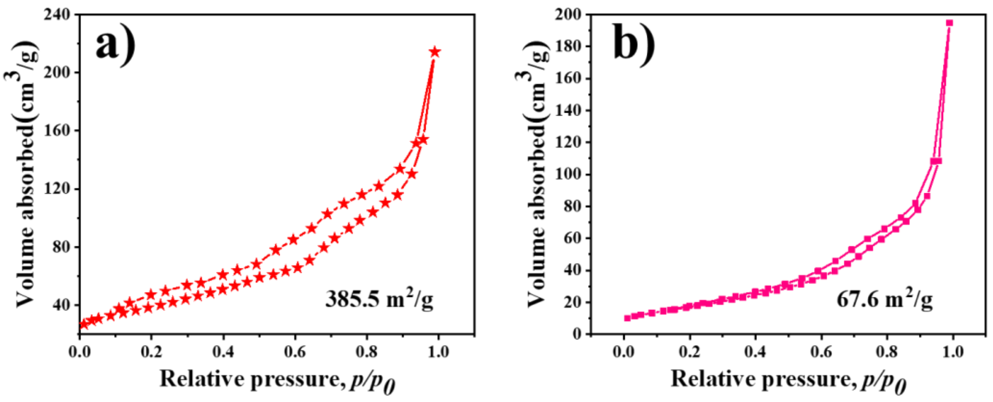

Supplementary Figure 5. N_2 adsorption–desorption isotherms curves of (a) 15@NSIs, and (b) NPs.

Supplementary Note 2. Composition analysis of P(VDF-HFP) polymer-based nanocomposite films

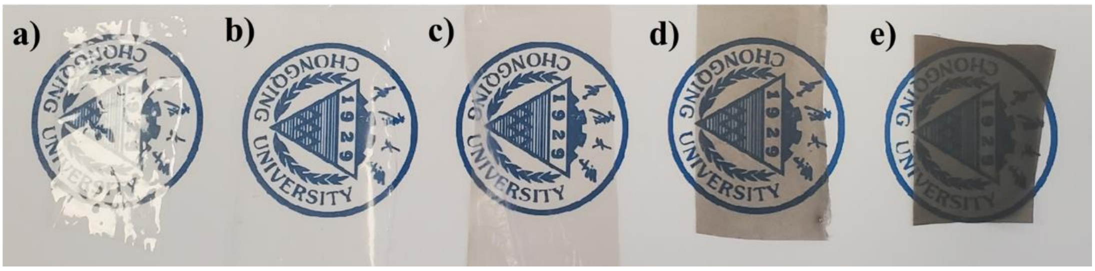

Supplementary Figure 6. Photographs of (a) pure P(VDF-HFP) film, (b) 15@NSIs-0.008wt%, (c) 15@NSIs-0.08wt%, (d) 15@NSIs-0.8wt%, and (e) 15@NSIs-3wt%. X@NSIs-Ywt% (X denoted as pore size, and Y denoted as doping amount), substrate designation is omitted.

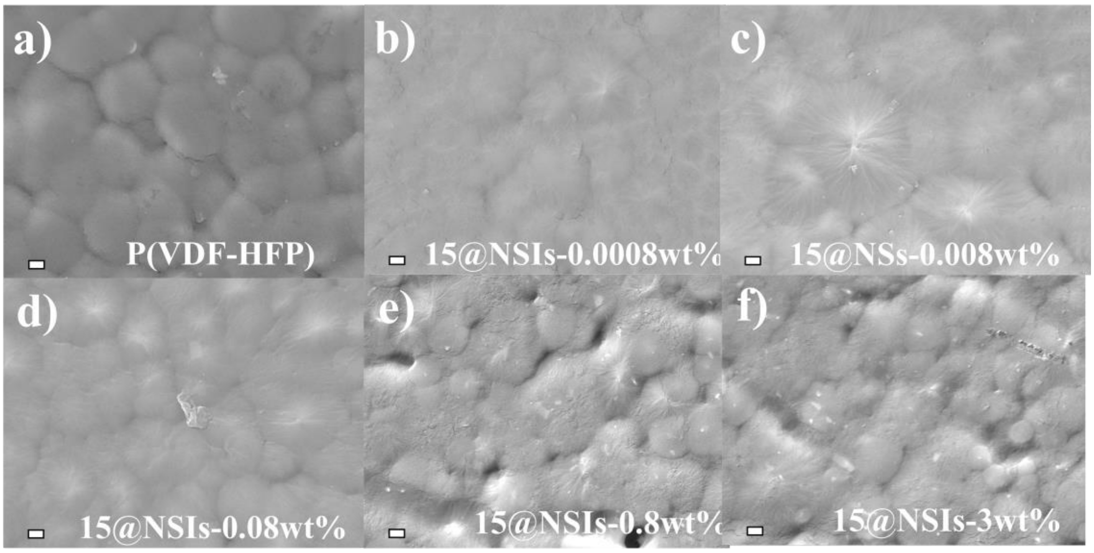

Supplementary Figure 7. The surface SEM images of (a) pure P(VDF-HFP) film, (b) 15@NSIs-0.0008wt%, (c) 15@NSIs-0.008wt%, (d) 15@NSIs-0.08wt%, (e) 15@NSIs-0.8wt%, and (f) 15@NSIs-3wt%. The scale bar is 1um.

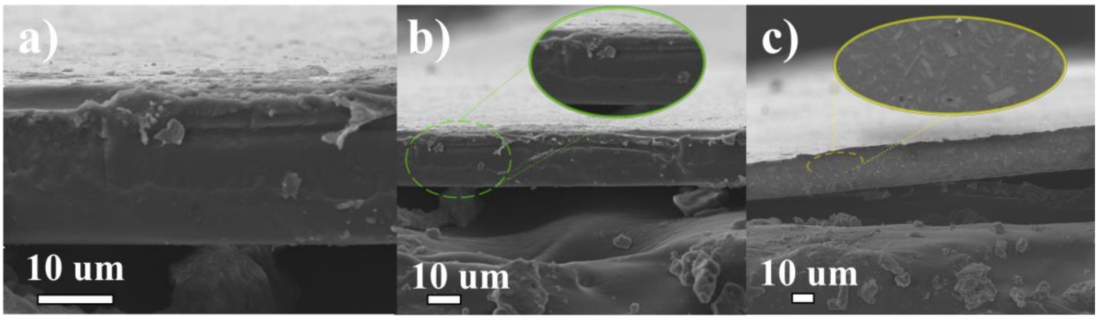

Supplementary Figure 8. Cross-sectional SEM images of (a) pure film, (b) 15@NSIs-0.008wt%, and 15@NSIs-0.008wt%.

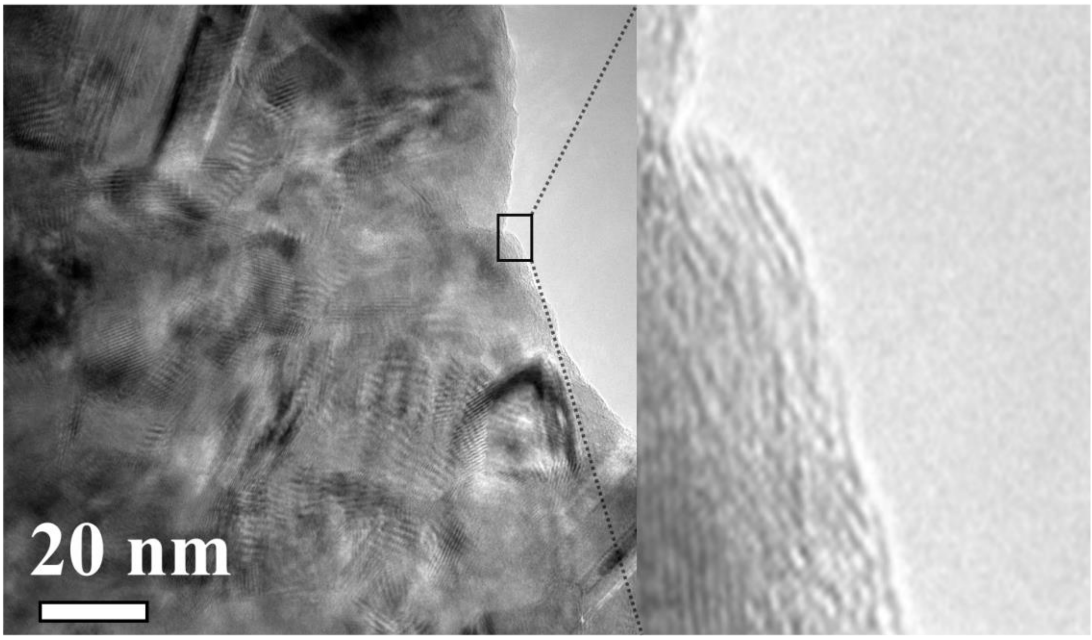

Supplementary Figure 9. HRTEM image of pure film (a) and its edges magnify image (b). Molecular segments of P(VDF-HFP) molecules, four to eight nm long, dominate the image in (b). The center-to-center distance varied around the ~0.425 nm distance between adjacent P(VDF-HFP) molecules.

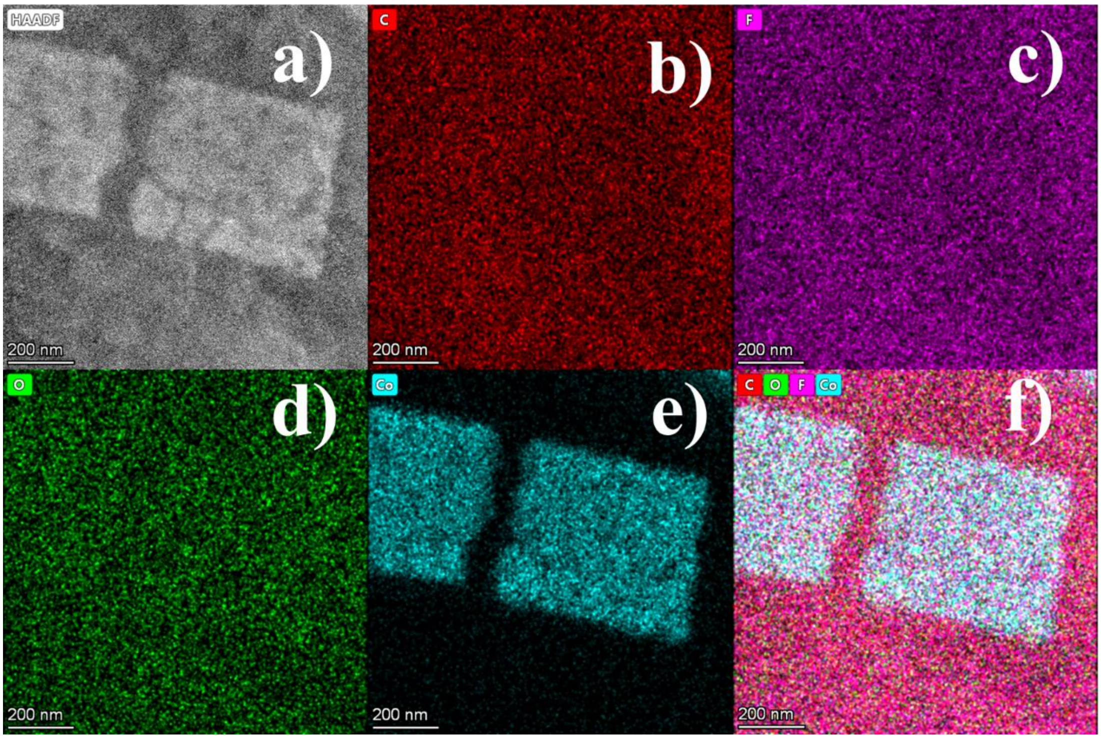

Supplementary Figure 10. (a) HAADF-STEM image of 15@NSIs-3wt%. HAADF-STEM-EDX elemental mapping images showing the element distributions of b) C, c) F, d) O and e) Co. The carbon is oxidized during the operation of Ar ion beam milling.

There are three main crystal structures of P(VDF-HFP), namely α, β and γ phases. The crystal planes corresponding to the diffraction peaks at 2θ=17.7°, 18.4°, 19.9°, and 26.8° are (100), (020), (110), and (021), respectively, which are all α crystal phases. The overlapping peaks corresponding to crystal planes (110) and (200) at around 2θ = 20.4° belong to the β crystal phase. The γ crystal diffraction peaks locate at 19.2° and near 20.2° corresponding (002) and (110)/ (101) facets.[1,2]
The relative content of α-, β- and γ-phase is calculated based on its characteristic absorbance bands of FTIR. The first step is to calculate the relative α-phase content by the ratio of non-polar and polar phases (α is non-polar, β and γ are polar). The specific equation is as follows:[3]

$$F_{\alpha} = \frac{A_n}{A_n + \left(\frac{K_n}{K_p}\right) A_p} \times 100\% \qquad (1)$$

Where F_{α} is the relative fraction of polarity, A_n and A_p are the absorbencies at 764 and 836 cm^{-1}, respectively. K_n and K_p represent the absorption coefficients: 6.1×10^4 and 7.7×10^4 cm^2/mol at the respective wavenumbers. The ratio of β- and γ-phase are further calculated through a similar method using the absorbance of their characteristic bands at 510 and 811 cm^{-1}, respectively.

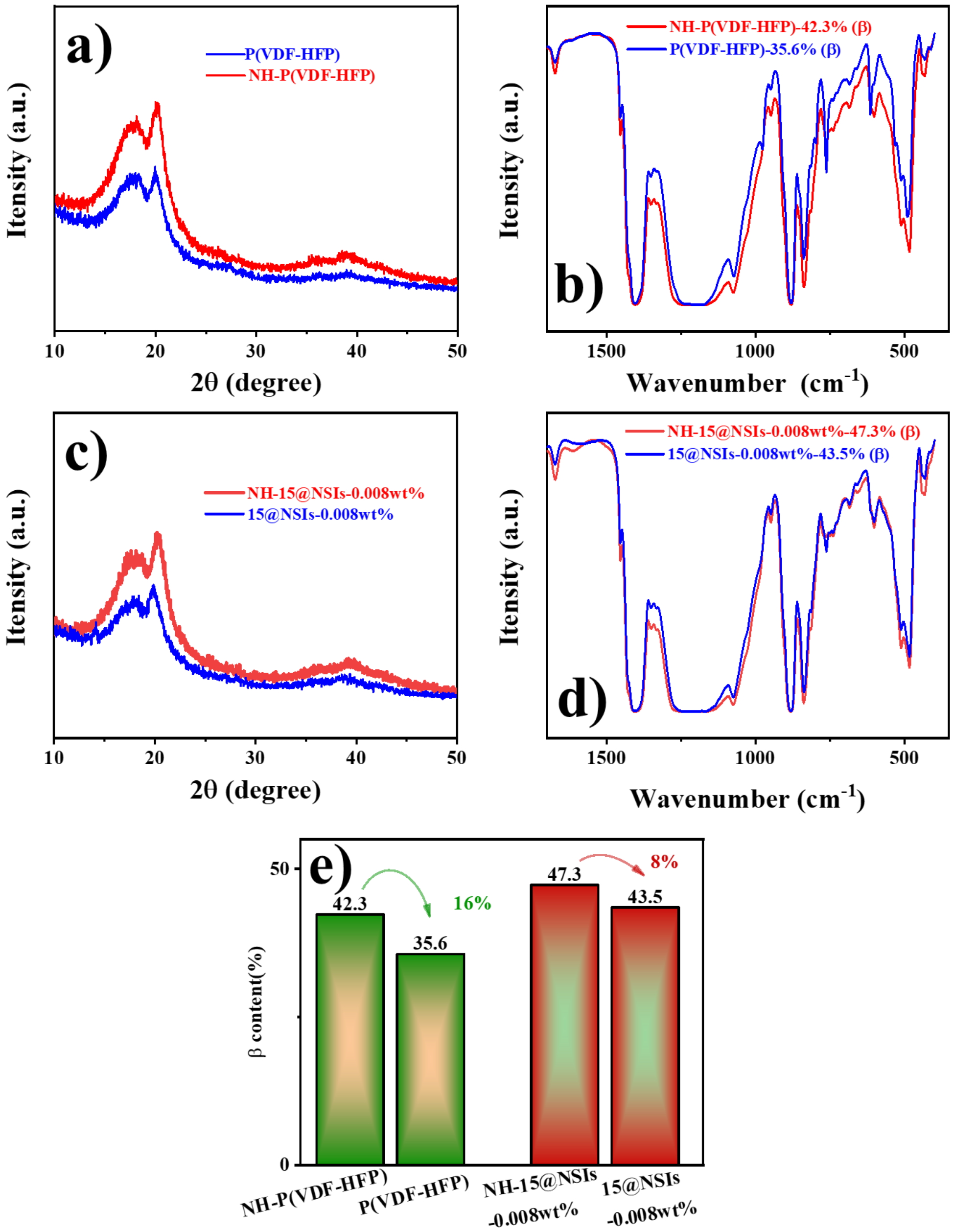

Supplementary Figure 11. (a-b) XRD patterns and Fourier-transform infrared (FTIR) spectra of no heat-treatment P(VDF-HFP) film (red line) and pure P(VDF-HFP) film (blue line). (c-d) XRD patterns and FTIR spectra of no thermal-treatment P(VDF-HFP) film (red line) and pure P(VDF-HFP) film (blue line). (e) Changes of β-phase content before and after heat treatment of pure film and 15@NSIs-0.008wt% nanocomposites.

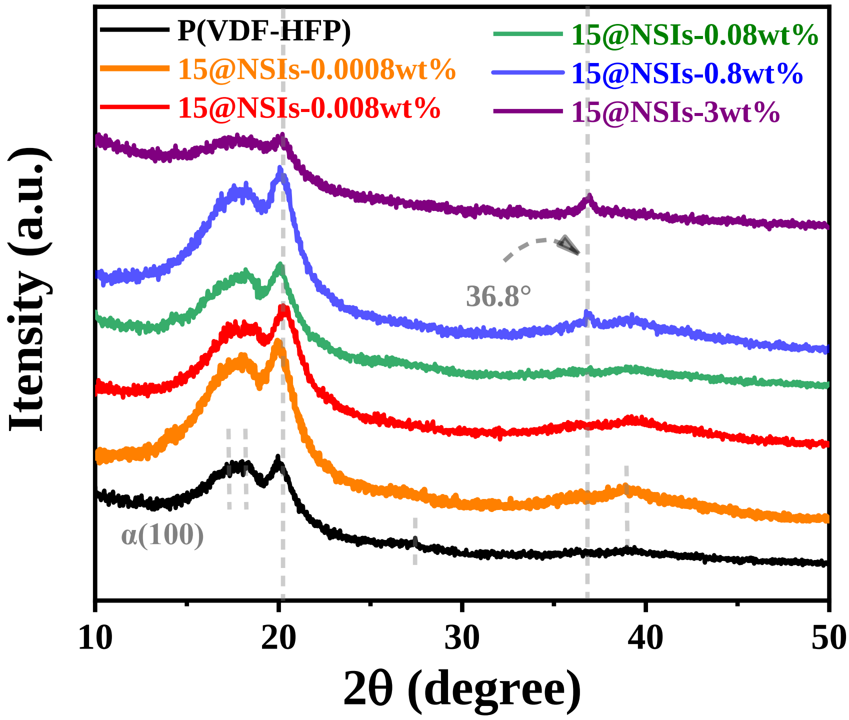

Supplementary Figure 12. XRD patterns of pure P(VDF-HFP) film, 15@NSIs-0.0008wt%, 15@NSIs-0.008wt%, 15@NSIs-0.08wt%, 15@NSIs-0.8wt%, and 15@NSIs-3wt%. 2θ=36.8° is Co_3O_4 characteristic peak.

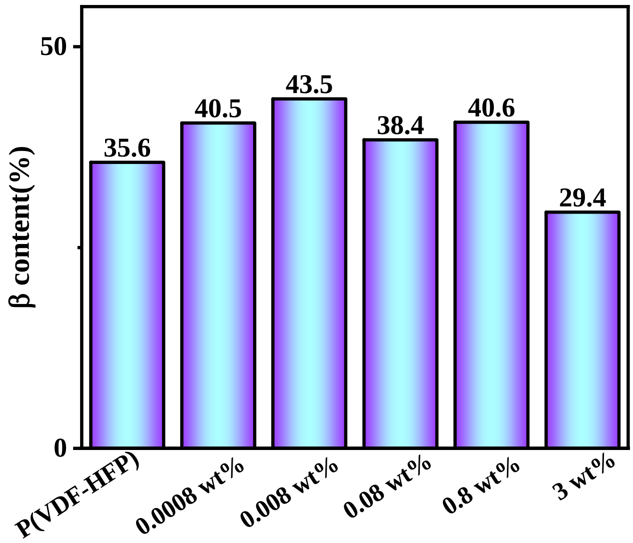

Supplementary Figure 13. Histogram of β content-doping fraction.

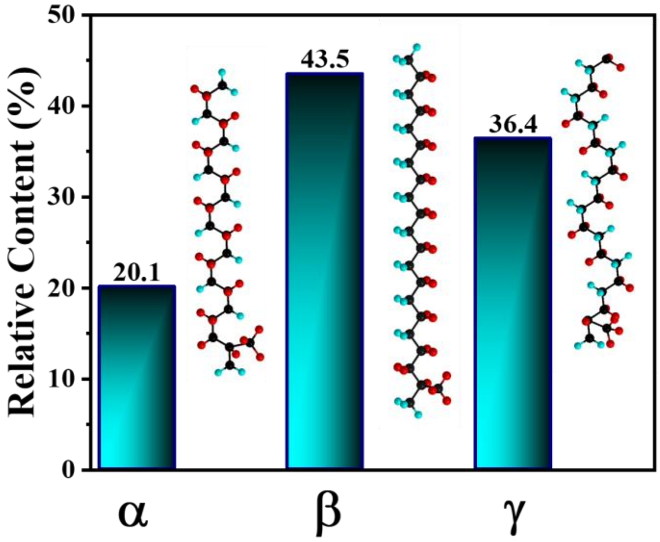

Supplementary Figure 14. The relative content and scheme illustration of α-, β-, and γ-phase in 15@NSIs-0.008wt%. the sphere colored by black, red, and light blue represent carbon, fluorine and hydrogen atoms, respectively.

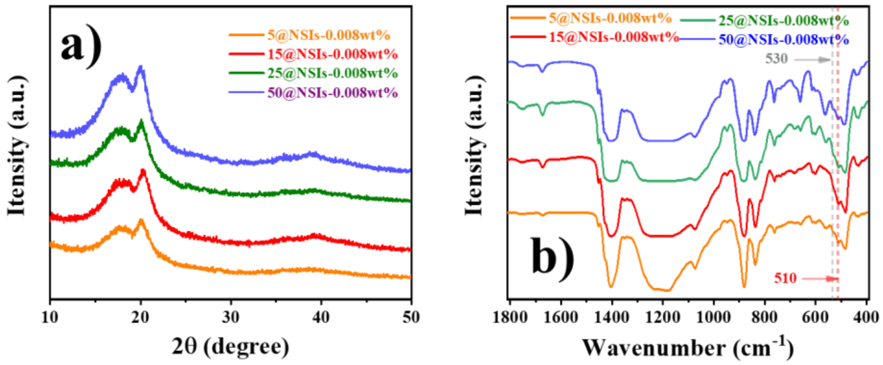

Supplementary Figure 15. XRD patterns (a) and FTIR spectra (b)5@NSIs-0.008wt%, 15@NSIs-0.008wt%, 25@NSIs-0.008wt%, and 50@NSIs-0.008wt%.

Supplementary Table 1. Relative fractions of α, β and γ conformations of P(VDF-HFP) and Co_3O_4 NSIs/P(VDF-HFP) nanocomposites.

Sample	TGTG'(α)%	TTTT(β)%	T_3GT_3G'(γ)%
P(VDF-HFP)	33.7	35.6	30.7
NH- P(VDF-HFP)	20.9	42.3	36.8
15@NSIs-0.0008wt%	25.7	40.5	33.8
NH-15@NSIs-0.008wt%	13.7	47.3	39.0
15@NSIs-0.008wt%	20.1	43.5	36.4
15@NSIs-0.08wt%	25.5	38.4	36.1
15@NSIs-0.8wt%	32.7	40.6	26.7
15@NSIs-3wt%	45.9	29.4	24.7
5@NSIs-0.008wt%	16.1	45.2	38.7
25@NSIs-008wt%	17.4	46.3	36.3
50@NSIs-0.008wt%	17.4	43.7	38.9

Supplementary Note 3. effect of nano fillers on electrical performances of P(VDF-HFP)

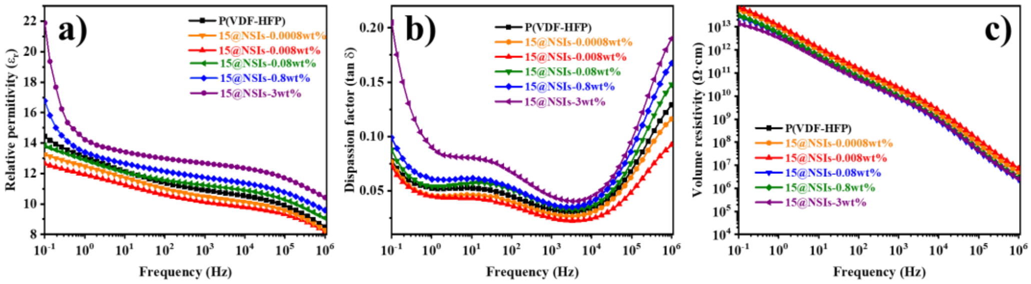

Supplementary Figure 16. Frequency dependence (0.1 Hz to 1 Hz) of dielectric constant (a), dispassion factor (b), and volume resistivity (c) of P(VDF-HFP) and 15@NSIs/P(VDF-HFP) with different filler contents.

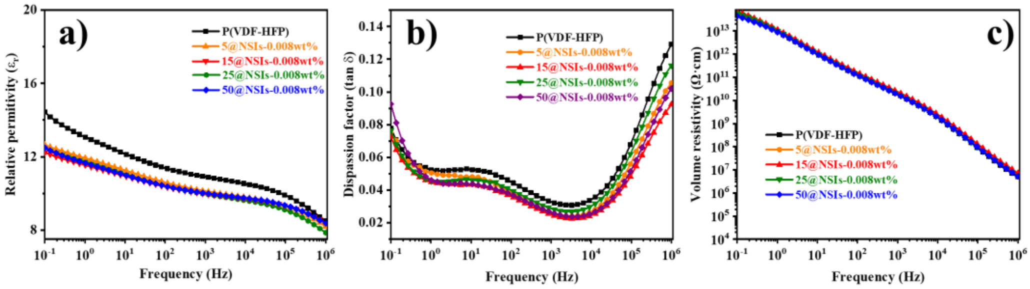

Supplementary Figure 17. Frequency dependence (0.1 Hz to 1 Hz) of dielectric constant (a), dispassion factor (b), and volume resistivity (c) of P(VDF-HFP) and X@SIs-0.008wt% with different pore size (X=5, 15, 25, and 50 nm).

The E_b of film is determined according to two parameters Weibull statistical analysis. the cumulative possibility of electric failure at measured electric field (E) is calculated according to the following formula:

$$P(E) = 1 - \exp\left\{-\left(\frac{E}{E_b}\right)^{\beta'}\right\} \tag{2}$$

Where E is the measure electric field at breakdown failure, E_b is the characteristic breakdown strength corresponding to an ≈63% probability failure, and β' is a shape parameter to evaluate the data scatter.

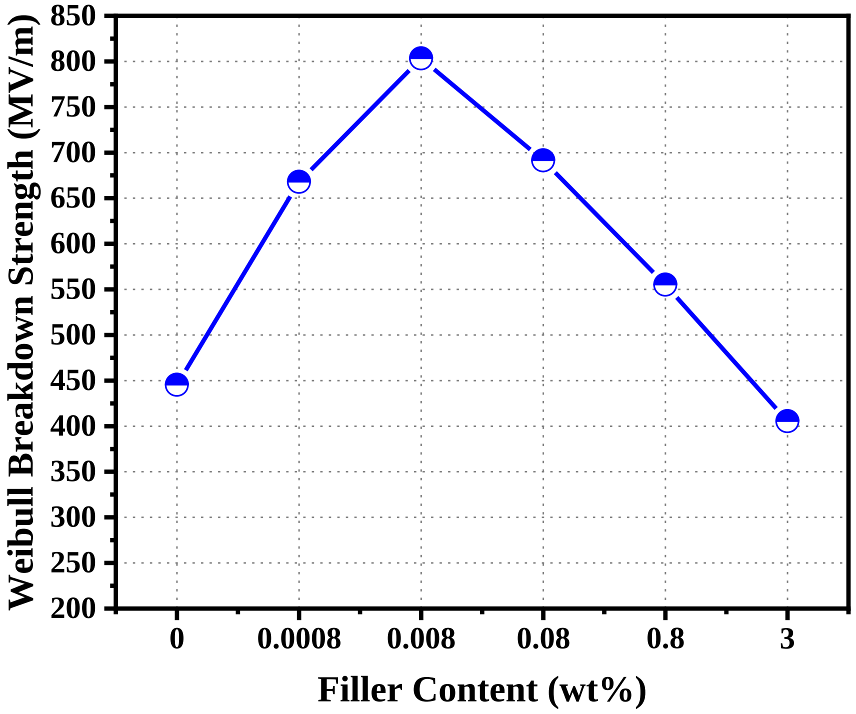

Supplementary Figure 18. Weibull breakdown strength E_b of 15@NSIs/P(VDF-HFP) as a function of the filler content.

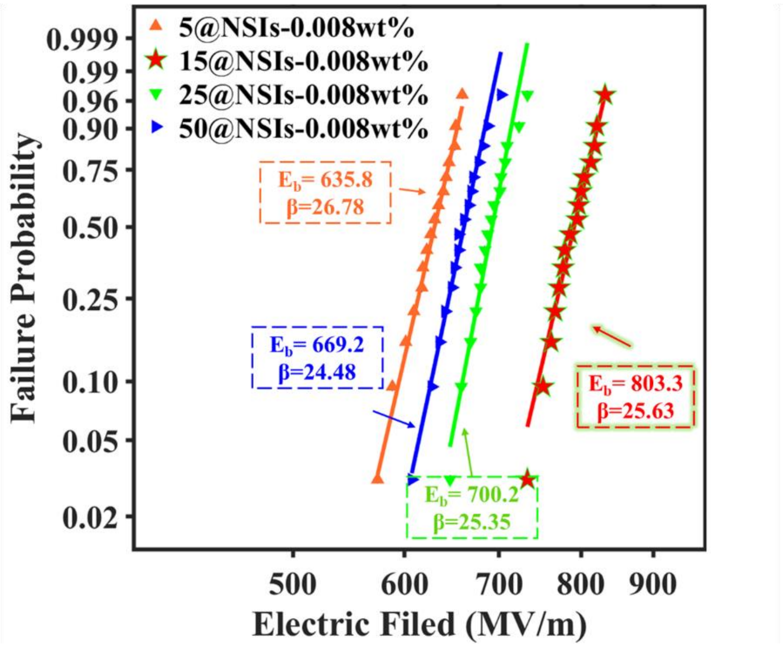

Supplementary Figure 19. Weibull plots of X@NSIs/P(VDF-HFP) with various pore sizes (X=5, 15, 25, and 50).

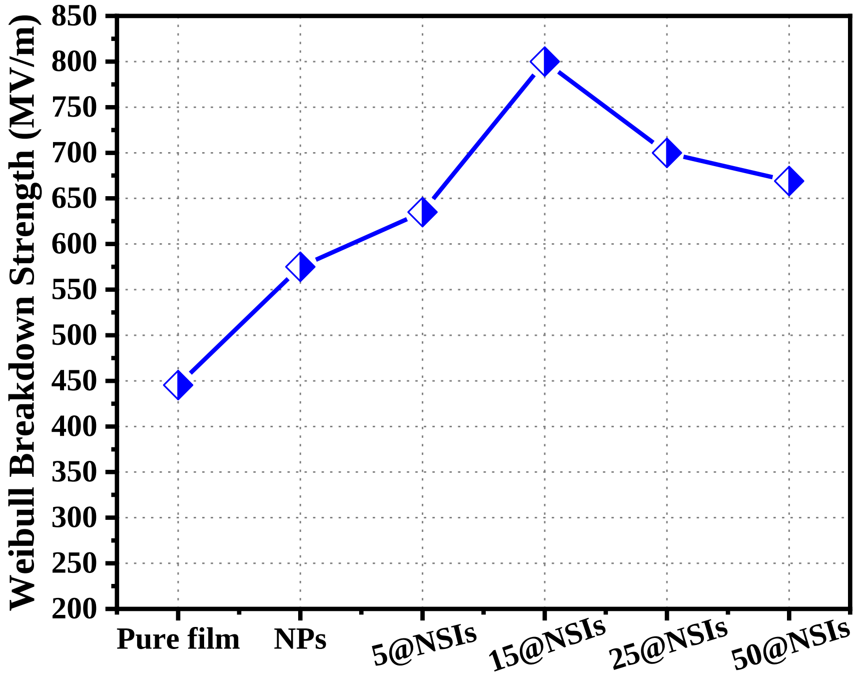

Supplementary Figure 20. Weibull breakdown strength E_b of X@NSIs/P(VDF-HFP) with various pore sizes (X=5, 15, 25, and 50).

Supplementary Note 4. electrostatic film capacitive energy storage properties

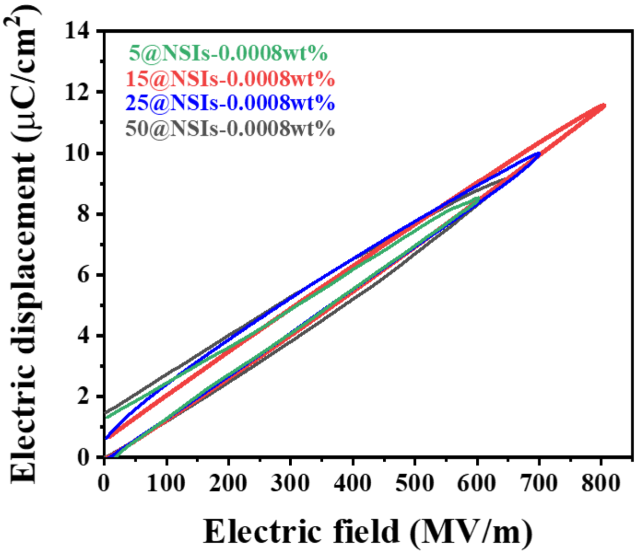

Supplementary Figure 21. Unipolar D-E loops of X@NSIs/P(VDF-HFP)-0.008wt% with different pore sizes (X=5, 15, 25, and 50 nm).

Supplementary Table 2. Comparison of the electric breakdown strength (E_b) and its enhancement ratio ($\Delta E_b/E_{b0}$) between this work and other literature.

Polymer	Filler	Filling ratio	$\Delta E_b/E_{b0}$ (%)	U_e (J cm^{-3})	η(%)	Ref.
P(VDF-HFP)	$Hf_{0.5}Zr_{0.5}O_2$	3 wt%	28	21.63	65.7	4
P(VDF-HFP)	BTO@TO_nfs	3 vol%	7.8	31.2	78.0	5
P(VDF-HFP)	LZO	15 vol%	-17	15.8	-	6
P(VDF-HFP)	BNNSs	9 wt%	18	10	70.0	7
P(VDF-HFP)	$(Bi_{0.5}Na_{0.5})$ TiO_3–$NaNbO_3$	0.5 wt%	17.5	36.94	63.0	8
P(VDF-HFP)	Ag-OMMT	4 vol%	21	10.51	80.0	9
P(VDF-HFP)	Al_2O_3 NPs	5 vol%	41.5	15.5	70.9	10
PPP(VDF-HFP)	Al_2O_3 NPLs	5 vol%	62.8	21.6	83.4	10
P(VDF-HFP)	TiO_2 _nfs	3 vol%	14	24	-	5
P(VDF-HFP)	Ag NDs	0.05 vol%	15	26.6	77.0	11
P(VDF-HFP)	CZS NDS	0.8 vol%	38	26	70.2	2
PVDF	BTO@TO nfs	3 vol%	7.8	17.5	75.0	12
PVDF	BNNS	10 vol%	37.5	13	58.0	13
**P(VDF-HFP)**	**15@Co_3O_4 NSIs**	**0.0008 wt%**	**50.1**	**20.2**	**84.0**	
**P(VDF-HFP)**	**15@Co_3O_4 NSIs**	**0.008 wt%**	**80.5**	**41.6**	**88.4**	
**P(VDF-HFP)**	**15@Co_3O_4 NSIs**	**0.08 wt%**	**55.4**	**26.7**	**69.2**	
**P(VDF-HFP)**	**15@Co_3O_4 NSIs**	**0.8 wt%**	**24.7**	**9.37**	**31.1**	
**P(VDF-HFP)**	**5@ Co_3O_4 NSIs**	**0.008 wt%**	**43.0**	**21.7**	**82.1**	
**P(VDF-HFP)**	**25@ Co_3O_4 NSIs**	**0.008 wt%**	**57.3**	**29.79**	**82.5**	
**P(VDF-HFP)**	**50@ Co_3O_4 NSIs**	**0.008 wt%**	**50.3**	**23.6**	**80.0**	

* NPs, NSs, nfs, and NDs represent nanoparticles, nanosheets, nanofibers, nanodots, respectively; BTO, TO, LZO, OMMT, and CZS represent $BaTiO_3$, TiO_2, $La_2Zr_2O_7$, organically modified montmorillonite, and $Cd_{1-x}Zn_xSe_{1-y}S_y$, respectively. The bolded part is the content of this work.

Supplementary Note 5. Density functional theory (DFT) calculations for the interface region in external strong electric field

All DFT calculations are performed with the Vienna ab initio package (VASP).[14-16] Spin polarization was applied in all the calculations within the framework of DFT, using the PBE exchange-correlation functional,[17] with the dispersion interaction corrected by the D3 scheme.[18] The cutoff energy for the plane waves is 400 eV, and the atomic core region is described by PAW pseudopotentials.[19] The DFT + U method using the Dudarev approach[20] was employed for the transition metal oxides. The U here is the effective Hubbard U_{eff}=U-J, where J is equal to zero and U is 3.0eV for the Co.[21,22] For the surface calculation, dipole correction and potential correction along the z-direction are also taken into consideration.[23] A 3×4×1 k-point mesh is used.

The experimental lattice constant of Co_3O_4 with 8.065Å is used in the calculation.[24] Co_3O_4(110) planes contain two different surface terminals which are donated as Co_3O_4(110)-A and Co_3O_4(110)-B. Co_3O_4(110)-A is more stable than Co_3O_4(110)-B.[25,26] Co_3O_4(110)-A includes the units of $Co_2^{2+}Co_2^{3+}O_4$; Co_3O_4(110)-B is comprised with $Co_2^{3+}O_4$. Co_3O_4(110)-A is modeled by the 8 layers, with the bottom 4 layers fixed in positions in the optimization. The lengths of a and b are 8.065 and 5.70 Å with a 15 Å vacuum layer along c. The adsorption energy is defined as

$$E_{ads} = E_{total} - E_{surface} - E_{m} \tag{3}$$

Where E_{total}, $E_{surface}$, and E_{m} are the energies of the system with molecule adsorption on the surface, the clean surface and the molecule, respectively.

For charge density difference alone z axis calculation, charge density difference ρ_{ads} is get first which is defined as

$$\rho_{ads} = \rho_{total} - \rho_{surface} - \rho_{m} \tag{4}$$

Where ρ_{total}, $\rho_{surface}$, and ρ_{m} are the charge on the system with molecule adsorption on the surface, the clean surface and the molecule, respectively. Then, we get charge density difference alone z axis.

For charge transfer calculation[27], the Bader population analysis is used to get charge on the system.

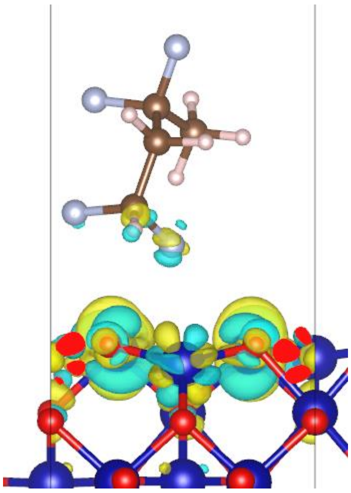

Supplementary Figure 22. The calculated charge density difference between polymer and Co_3O_4.

Supplementary Table 3. The table for the strength of internal electric field, charge on the $Co_3O_4(110)$ surface, and the distance between F atom and Co^{3+} with molecule adsorption on Co^{3+} site.

Electric field (eV/ Å)	Δ E (eV)	Charge on surface (|e|)	d_1(Å)	d_2(Å)
0.00	12.34	0.021	2.602	2.885
-0.06	12.49	0.019	2.598	2.884
-0.03	12.41	0.020	2.600	2.884
0.03	12.27	0.022	2.606	2.888
0.06	12.41	0.024	2.585	2.922

Supplementary Table 4. Interface charge transfer under different E_{ex}. (Co_3O_4→P(VDF-HFP) molecular is positive direction)

Electric field (eV/ Å)	Charge transfer (μC/m2)	Charge transfer ratio (%)
0.00	0.0073	0
-0.06	0.0066	-9.59
-0.03	0.0070	-4.11
0.03	0.0077	5.48
0.06	0.0083	13.70

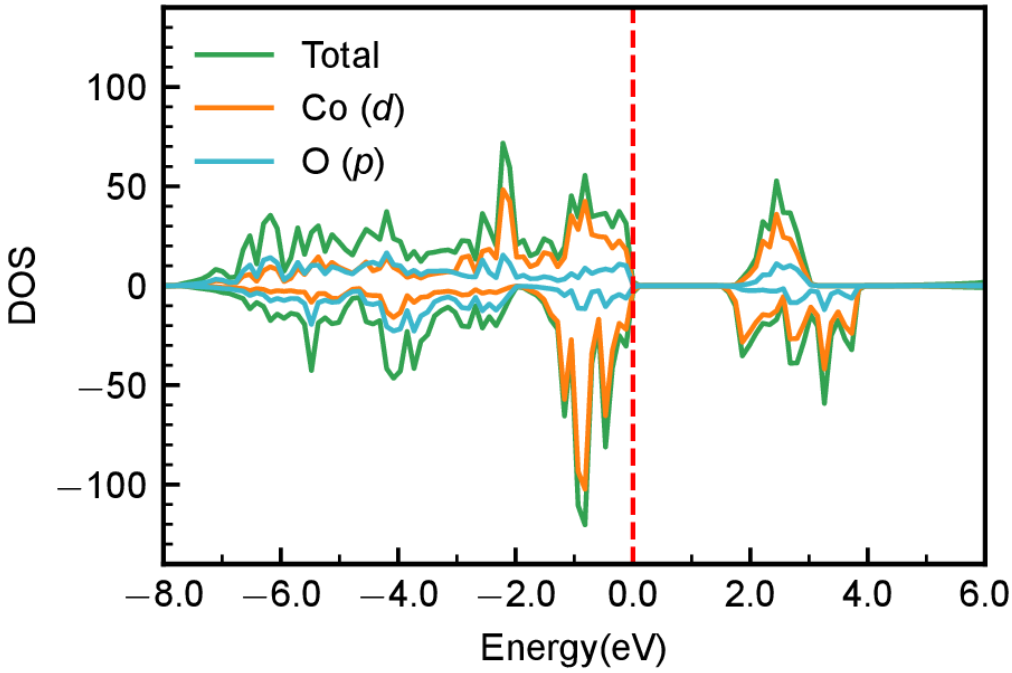

Supplementary Figure 23. Density of states of bulk Co_3O_4. Red dash line represents the fermi level..

Supplementary Note 6. Macro-finite element simulation (FES) revised by DFT results to analyze the mechanism of nano-fillers

At a given operating field, the electric field (E) and electric potential (φ) in the P(VDF-HFP)-based nanocomposites films can be expressed as following specialized Maxwell's equations.

$$\Delta \cdot (\varepsilon_0 K E) = \rho$$
$$\Delta \cdot J = \frac{-\partial \rho}{\partial t} \qquad (5)$$
$$E = -\nabla \varphi$$
$$J = \sigma E$$

Where ε_0 is vacuum dielectric constant (≈8.854187817×10^{-12} F/m), K is relative dielectric constant, E is electric field, ρ is space charge density, J is current density, φ is electric potential, σ is electrical conductivity.

E distortion is highly detrimental to capacitors if its degree is overlarge because it degrades not only the dielectric performance, but also the device reliability and service lifetime. In this section, based on the result of DFT that the intermolecular charge transfer in Co_3O_4-P(VDF-HFP) inhibits the polariton of interfacial polymer molecules at high E, we revise the interface region parameters in model and simulate the steady-state internal E and φ distribution in nanocomposites by using finite element computations. Electric field modeling is complicated for nano-modified polymer matrix composites because there is significant variability in materials' properties, which are determined by temperature, orientation, crystallinity, etc. Note that the focus of this work is development of high electric field strength resistant insulation materials. The simulation involved in this study is simplified with several assumptions being made: 1) The samples are placed in an electric field of 200MV/m to explore the partial E distortion and potential distribution around the single nanoparticles, nanosheets, or nano-sieves. 2) 2D nanofillers are placed horizontally perpendicular to the direction of the applied electric field. 3) the polymer substrate is supposed to be homogeneous everywhere, with no distinction between crystalline and amorphous regions. 4) the parameters of nanofillers' properties are constant overtime in each case such that the partial electrical field and potential of dielectrics are only a function of morphology of nanofillers. 5) The K and σ values of components in nanocomposites stays unchanged in the simulation. The details of the simulation are discussed in the rest part of this section.

The radius of nanoparticles is 8 nm. In order to make the obtained images present better visual effects, we set the length and width of the 2D nanomaterials to 200×500 nm. The specific model figures are shown in Supplementary Figure 21. The dielectric constant (ε) of P(VDF-HFP) and Co_3O_4 are set to be 10 and 20, respectively.

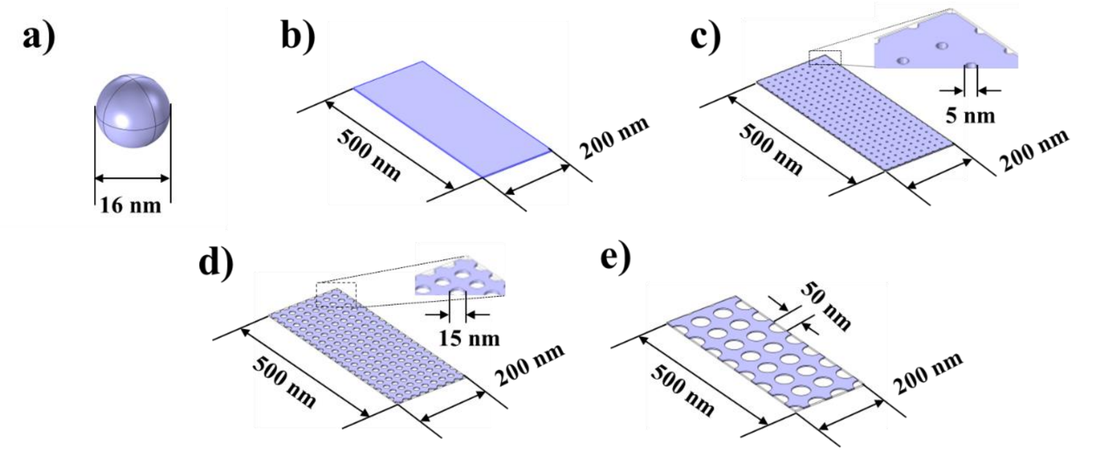

Supplementary Figure 24. The model of Co_3O_4 (a) nanoparticle, (b) nanosheet, (c) nano-sieve with 5 nm pore size, (d) nano-sieve with 15 nm pore size, and (e) nano-sieve with 50 nm pore size.

In contrast to the routine COMSOL electric field simulation of nanoparticles-modified dielectrics that only consider two parts, fillers and substrates, does not in-depth consider about the interface region, which is generally recognized to be the part that plays a key role in enhancing the electrical performance of the dielectrics. Thus, a shell layer 0.5 nm thick is coated on the surface of the nanofillers to represent the interfacial region where the polarization of the polymer molecule is affected according to the results of first-principles calculations in the section 5. The K of upper interface layer (UIL) is set to 14, the K of lower interface layer (LIL) is set to 10. This is a rough qualitative correction. As shown in Supplementary Fig. 25a and b, it is well-observed that the electric potential lines are more intensive on the upper and lower sides of nanoparticles because of the mis-match in K values of the fillers and polymer matrix, which results in the concentrated electric field on the filler-polymer interfaces. In the simulation result (Supplementary Fig. 25c) without interfacial layer, the E distortion on the upper and lower surfaces caused by nanoparticles is symmetric. But, in the simulation plot (Supplementary Fig. 25d) with interfacial layer correction, the E distortion on the upper and lower surfaces caused by nanoparticles is asymmetric. The enhanced polymer molecular polarization of UIL achieves to a better buffer matching in filler-polymer, which results in a reduced degree of E distortion. This might be essential to achieve increased breakdown strength of dielectric nanocomposites. Therefore, the breakdown mechanism of nano-modified dielectrics could be explicated more reasonably by using interfacial layer correction method.

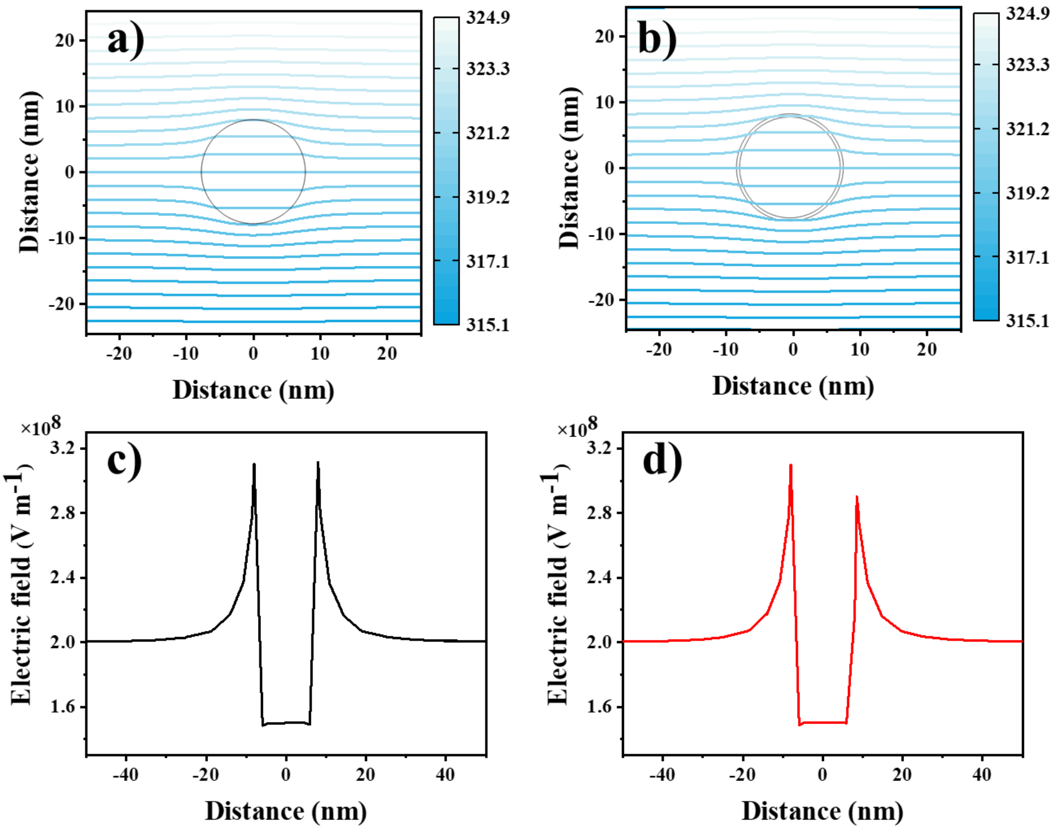

Supplementary Figure 25. Simulated electric potential lines of (a) nanoparticle without interfacial layer and (b) nanoparticle with interfacial layer correction in P(VDF-HFP) dielectric. Simulated local electric field (vertical direction) near a nanoparticle/polymer matrix interface of (c) nanoparticle without interfacial layer and (d) nanoparticle with interfacial layer correction in P(VDF-HFP) dielectric under an external E=200MV/m.

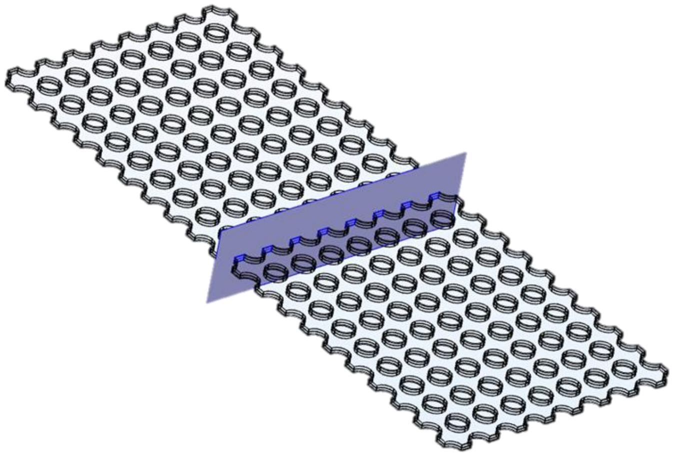

Supplementary Figure 26. A 60×220 nm vertical cross-section region at the center of the NSIs.

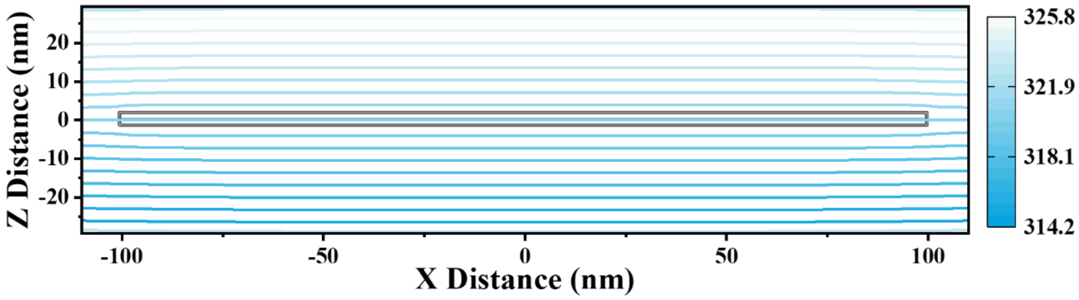

Supplementary Figure 27. Simulated local electric potential lines around the nanosheet in P(VDF-HFP) dielectric.

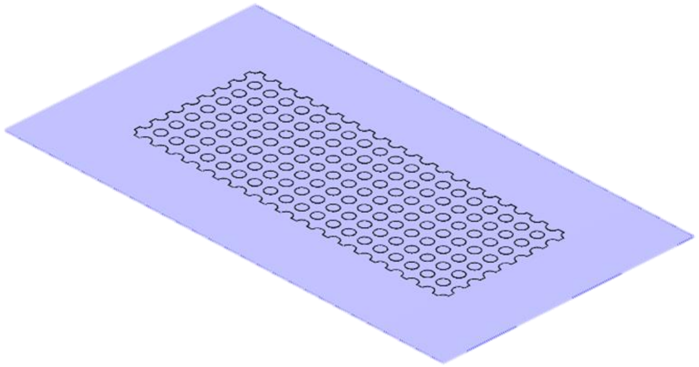

Supplementary Figure 28. A 400×700 nm horizontal cross-section surface 0.5 nm away from the interface layer.

As shown in Fig. S26, from the column on the left, there are some points can be obtained. NPs/P(VDF-HFP) generate a downward conical potential trap with a minimum value obtained at the position of the center of the circle corresponding to the nanoparticle. Nanosheet has a concave basin-like potential trap. When 5nm pores appeared on the nanosheet, some upward protrusions began to appear at the bottom of the concave basin. As the pore size increases to 15nm, the bumps grow upwards, forming what resembles a tubular array. When the pore size reaches the maximum value of 50 nm, the protrusions grow further and are almost equal to the theoretical potential. When the pore diameter reaches a maximum value of 50 nm, the protrusions grow further almost equal to V_{theory}, but the size limitation makes their quantities decrease sharply. The E distribution diagram on the right column also shows a similar pattern.

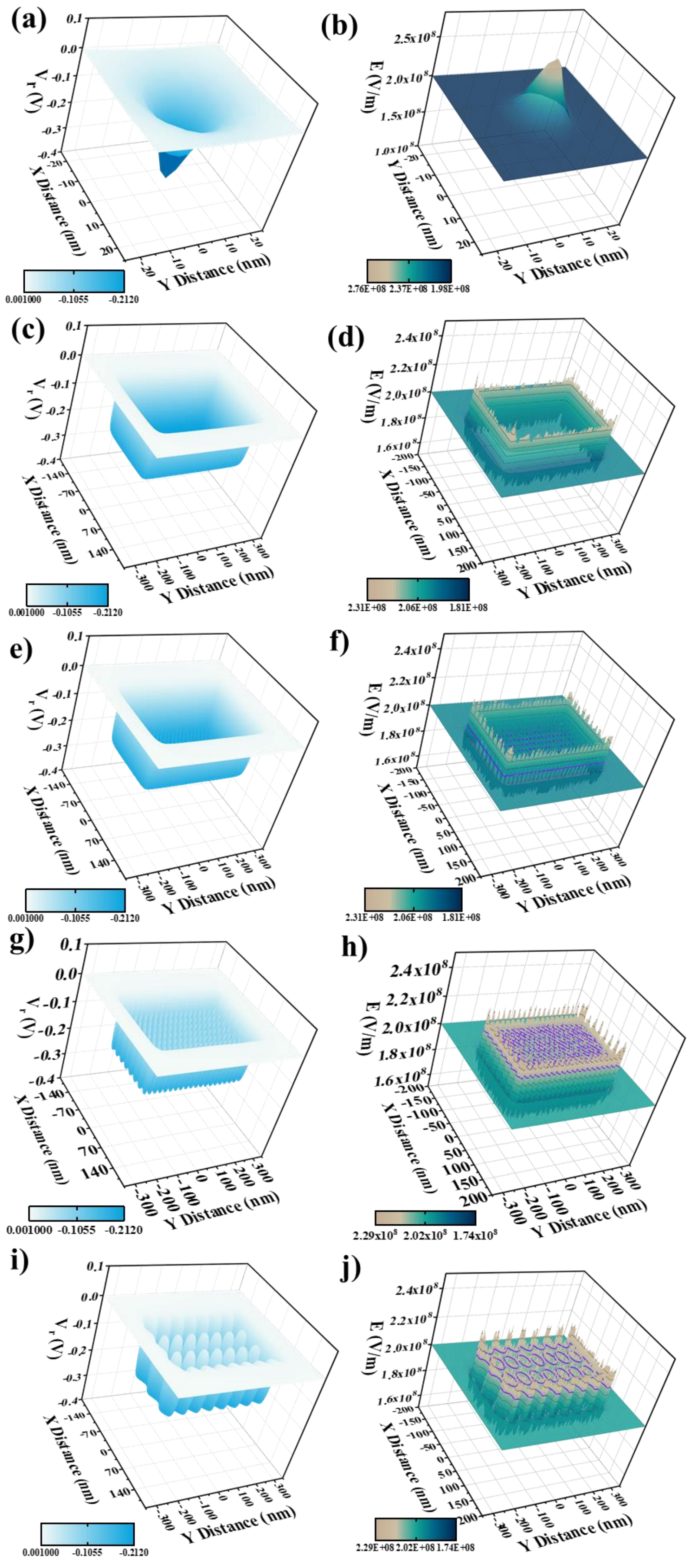
(a)
(b)
(c)
(d)
e)
f)
g)
h)
i)
j)
V_r (V)
E (V/m)
X Distance (nm)
Y Distance (nm)
0.001000 -0.1055 -0.2120
2.76E+08 2.37E+08 1.98E+08
2.31E+08 2.06E+08 1.81E+08
2.29x10⁸ 2.02x10⁸ 1.74x10⁸
2.29E+08 2.02E+08 1.74E+08

Supplementary Figure 29. 3D graphs of V_r of (a) nanoparticle, (c) nanosheet, (e) 5@NSIs, (g) 15@NSIs, and (i) 50@NSIs. 3D graphs of normE of (b) nanoparticle, (d) nanosheet, (f) 5@NSIs, (h) 15@NSIs, and (j) 50@NSIs. From the column on the left, there are some points can be obtained. NPs/P(VDF-HFP) generate a downward conical potential trap with a minimum value obtained at the position of the center of the circle corresponding to the nanoparticle. Nanosheet has a concave basin-like potential trap. When 5nm pores appeared on the nanosheet, some upward protrusions began to appear at the bottom of the concave basin. As the pore size increases to 15nm, the bumps grow upwards, forming what resembles a tubular array. When the pore size reaches the maximum value of 50 nm, the protrusions grow further and are almost equal to the theoretical potential. When the pore diameter reaches a maximum value of 50 nm, the protrusions grow further almost equal to V_{theory}, but the size limitation makes their quantities decrease sharply. The E distribution diagram on the right column also shows a similar pattern.

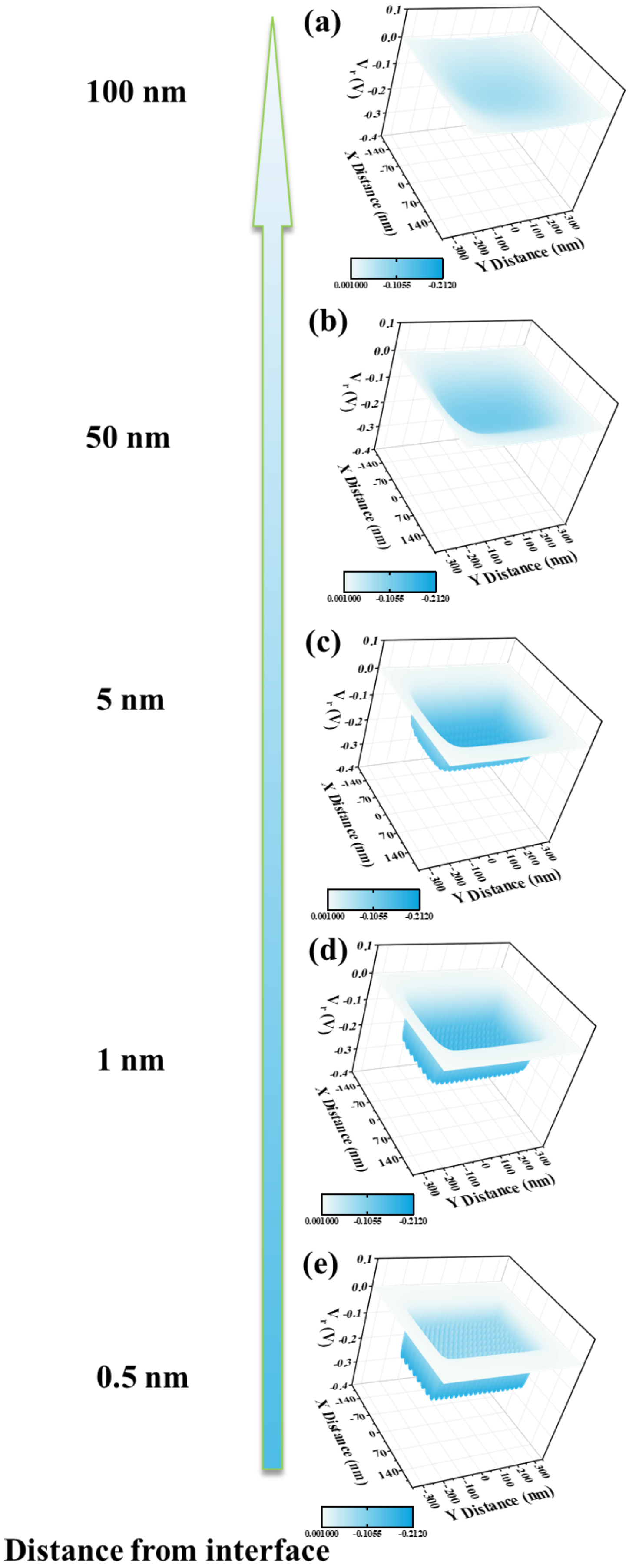
100 nm
50 nm
5 nm
1 nm
0.5 nm
Distance from interface
(a)
(b)
(c)
(d)
(e)
V_r (V)
X Distance (nm)
Y Distance (nm)
0.001000
-0.1055
-0.2120

Supplementary Figure 30. The planar potential distribution 3D graphs of V_r of 15@NSIs near the interface (a) 100nm, (b) 50 nm, (c) 5nm, (d) 1 nm, and (e) 0.5 nm. In the region far from the interface (>20 nm), the planar potential distribution exhibits a relatively weaker and wider pit trap. Its effect is similar to that of nanosheets. However, in the region of 0~10 nm, the planar potential shows a intricate 3D trap array distribution, reflecting the electric field cavity effect caused by the porous structure of the nano-sieve.

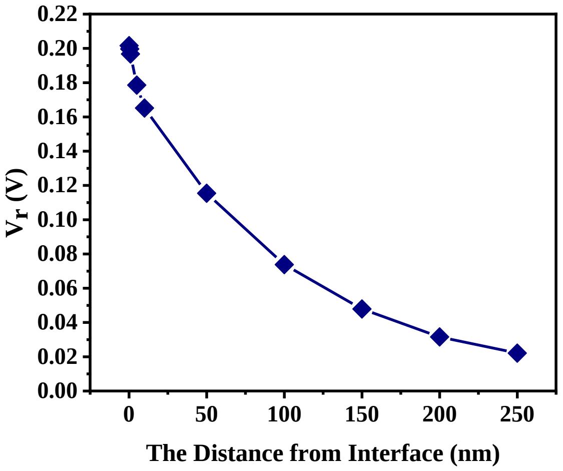

Supplementary Figure 31. The depth of potential trap (V_r) - the distance from the interface curve.

Supplementary References

1 Gregorio, R. & Ueno, E. M. Effect of crystalline phase, orientation and temperature on the dielectric properties of poly (vinylidene fluoride)(PVDF). *Journal of materials science* **34**, 4489-4500 (1999).

2 Li, L. *et al.* Significant improvements in dielectric constant and energy density of ferroelectric polymer nanocomposites enabled by ultralow contents of nanofillers. *Advanced Materials* **33**, 2102392 (2021).

3 García-Payo, M. d. C., Essalhi, M. & Khayet, M. Effects of PVDF-HFP concentration on membrane distillation performance and structural morphology of hollow fiber membranes. *Journal of Membrane Science* **347**, 209-219 (2010).

4 Chen, H. *et al.* Enhanced energy density in sandwich-structured P (VDF-HFP) nanocomposites containing Hf0. 5Zr0. 5O2 nanofibers. *Chemical Engineering Journal* **436**, 131123 (2022).

5 Zhang, X. *et al.* Giant energy density and improved discharge efficiency of solution-processed polymer nanocomposites for dielectric energy storage. *Advanced Materials* **28**, 2055-2061 (2016).

6 Mj, K. K. & Kalathi, J. T. Investigation on the dielectric performance of PVDF-HFP/LZO composites. *Journal of Alloys and Compounds* **843**, 155889 (2020).

7 Ma, G. *et al.* Improved dielectric and energy storage properties of polymer composites with BNNSs/AgNPs hybrid nanofiller. *Materials Technology*, 1-8 (2022).

8 Xie, X. *et al.* Ultralow-Content (Bi0. 5Na0. 5) TiO3–NaNbO3/PVDF-HFP Nanocomposites for Ultrahigh-Energy-Density Capacitor Applications. *ACS Applied Energy Materials* (2022).

9 Wang, H. *et al.* Enhanced dielectric property and energy storage density of PVDF-HFP based dielectric composites by incorporation of silver nanoparticles-decorated exfoliated montmorillonite nanoplatelets. *Composites Part A: Applied Science and Manufacturing* **108**, 62-68 (2018).

10 Li, H. *et al.* Enabling high-energy-density high-efficiency ferroelectric polymer nanocomposites with rationally designed nanofillers. *Advanced Functional Materials* **31**, 2006739 (2021).

11 Huang, X. *et al.* Non-intuitive concomitant enhancement of dielectric permittivity, breakdown strength and energy density in percolative polymer nanocomposites by trace Ag nanodots. *Journal of Materials Chemistry A* **7**, 15198-15206 (2019).

12 Zhang, X. *et al.* Ultrahigh energy density of polymer nanocomposites containing BaTiO3@ TiO2 nanofibers by atomic-scale interface engineering. *Advanced materials* **27**, 819-824 (2015).

13 Liu, F. *et al.* High-energy-density dielectric polymer nanocomposites with trilayered architecture. *Advanced Functional Materials* **27**, 1606292 (2017).

14 Kresse, G. & Furthmüller, J. Efficiency of ab-initio total energy calculations for metals and semiconductors using a plane-wave basis set. *Computational materials science* **6**, 15-50 (1996).

15 Kresse, G. & Furthmüller, J. Efficient iterative schemes for ab initio total-energy calculations using a plane-wave basis set. *Physical review B* **54**, 11169 (1996).

16 Kresse, G. & Hafner, J. Ab initio molecular dynamics for liquid metals. *Physical review B* **47**, 558 (1993).

17 Perdew, J. P., Burke, K. & Ernzerhof, M. Generalized gradient approximation made simple. *Physical review letters* **77**, 3865 (1996).

18 Grimme, S., Ehrlich, S. & Goerigk, L. Effect of the damping function in dispersion corrected

density functional theory. *Journal of computational chemistry* **32**, 1456-1465 (2011).
19 Kresse, G. & Joubert, D. From ultrasoft pseudopotentials to the projector augmented-wave method. *Physical review b* **59**, 1758 (1999).
20 Dudarev, S. L., Botton, G. A., Savrasov, S. Y., Humphreys, C. J. & Sutton, A. P. Electron-energy-loss spectra and the structural stability of nickel oxide: An LSDA+ U study. *Physical Review B* **57**, 1505 (1998).
21 Peng, Y., Hajiyani, H. & Pentcheva, R. Influence of Fe and Ni doping on the OER performance at the Co3O4 (001) surface: insights from DFT+ U calculations. *ACS Catalysis* **11**, 5601-5613 (2021).
22 Farkaš, B., Santos-Carballal, D., Cadi-Essadek, A. & De Leeuw, N. H. A DFT+ U study of the oxidation of cobalt nanoparticles: Implications for biomedical applications. *Materialia* **7**, 100381 (2019).
23 Neugebauer, J. & Scheffler, M. Adsorbate-substrate and adsorbate-adsorbate interactions of Na and K adlayers on Al (111). *Physical Review B* **46**, 16067 (1992).
24 Roth, W. L. The magnetic structure of Co3O4. *Journal of Physics and Chemistry of Solids* **25**, 1-10 (1964).
25 Yang, C., Zhao, Z.-Y. & Liu, Q.-J. Theoretical study of CO oxidation on Au1/Co3O4 (110) single atom catalyst using density functional theory calculations. *Materials Science in Semiconductor Processing* **123**, 105578 (2021).
26 Creazzo, F., Galimberti, D. R., Pezzotti, S. & Gaigeot, M.-P. DFT-MD of the (110)-Co3O4 cobalt oxide semiconductor in contact with liquid water, preliminary chemical and physical insights into the electrochemical environment. *The Journal of chemical physics* **150**, 041721 (2019).
27 Bader, R. F. W. Principle of stationary action and the definition of a proper open system. *Physical Review B* **49**, 13348 (1994)..
